# Babassu nut residues: potential for bioenergy use in the North and Northeast of Brazil

**DOI:** 10.1186/2193-1801-3-124

**Published:** 2014-03-06

**Authors:** Thiago de Paula Protásio, Paulo Fernando Trugilho, Antônia Amanda da Silva César, Alfredo Napoli, Isabel Cristina Nogueira Alves de Melo, Marcela Gomes da Silva

**Affiliations:** Departamento de Ciências Florestais, Universidade Federal de Lavras - UFLA, Câmpus Universitário s/n, Caixa Postal: 3037 Lavras, MG Brazil; Centre de Coopération Internationale En Recherche Agronomique Pour Le Dévelopment - CIRAD, Biomass, Wood, Energy, Bioproducts Unit (BioWooEB), Rue Jean-François Breton, 34398 Montpellier, France; Universidade Federal Rural da Amazônia – UFRA, Avenida Tancredo Neves, n. 2501, Caixa Postal: 917 Belém, PA Brazil

**Keywords:** Babassu, Characterization, Alternative biofuel, Charcoal, Biomass

## Abstract

Babassu is considered the largest native oil resource worldwide and occurs naturally in Brazil. The purpose of this study was to evaluate the potential of babassu nut residues (epicarp, mesocarp and endocarp) for bioenergy use, especially for direct combustion and charcoal production. The material was collected in the rural area of the municipality of Sítio Novo do Tocantins, in the state of Tocantins, Brazil. Analyses were performed considering jointly the three layers that make up the babassu nut shell. The following chemical characterizations were performed: molecular (lignin, total extractives and holocellulose), elemental (C, H, N, S and O), immediate (fixed carbon, volatiles and ash), energy (higher heating value and lower heating value), physical (basic density and energy density) and thermal (thermogravimetry and differential thermal analysis), besides the morphological characterization by scanning electron microscopy. Babassu nut residues showed a high bioenergy potential, mainly due to their high energy density. The use of this biomass as a bioenergy source can be highly feasible, given their chemical and thermal characteristics, combined with a low ash content. Babassu nut shell showed a high basic density and a suitable lignin content for the sustainable production of bioenergy and charcoal, capable of replacing coke in Brazilian steel plants.

## Background

Currently, the main countries’ concerns with energy are related to overuse and dependence on fossil fuels, to the dangers of CO_2_ emissions and concentration in the atmosphere, and to global warming (Zhu et al. 2011). According to estimates by IEA3 (International Energy Agency 2001), a 53% increase in energy consumption is expected in the world by 2035, and fossil fuels will provide most of the energy used. The consumption of renewable energy must increase from 10% in 2008 to 14% in 2035 (International Energy Agency 2001).

This shows the dependence of humanity on non-renewable fuels and the need for scientific research and technological development, in order to diversify energy sources and reduce the consumption of such fuels, thus contributing to the consolidation of a safer and less polluting energy matrix.

In this context, plant biomass has been considered a potential renewable energy source, which can greatly contribute to reducing the consumption of non-renewable fuels and therefore reduce greenhouse gas emissions (Sheng and Azevedo 2005; Moghtaderi et al. 2006; Shen and Gu 2009; Kim et al. 2010; Protásio et al. 2013a). The interest in the use of biomass as an alternative energy source is the fact that it is a sustainable and continuously regenerating material (Poletto et al. 2012). Furthermore, the energy use of lignocellulosic residues is a feasible alternative for sustainability and avoids the large-scale pollution of soil, water and air (Protásio et al. 2013a).

Although the world energy matrix is almost exclusively dependent on fossil fuels (International Energy Agency 2001), some countries have taken advantage of their agroforestry potential to increase the use of plant biomass as an alternative energy source (Protásio et al. 2013a; Wright 2006). This is the case of Brazil, in which 44.1% of the domestic energy supply comes from renewable sources, with a participation of 25.4% of the various biomass products (Empresa de Pesquisa Energética – EPE 2012).

The country is one of the few that has great potential to expand the use and production of biomass due to the wide availability of growing areas, as well as lignocellulosic residues from the agricultural industry (Rousset et al. 2013; Dias et al. 2012). Sugar cane, with 9,616,615 ha (Instituto Brasileiro de Geografia e Estatística 2011) and *Eucalyptus*, with 4,873,952 ha (Associação Brasileira de Produtores de Florestas Plantadas – ABRAF 2012) are the two main sources of biomass for energy in Brazil. However, there are other lignocellulosic materials with high potential for bioenergy use, such as babassu nut, with 14,563,000 native ha (Empresa Brasileira de Pesquisa Agropecuária 1984; Teixeira 2008).

Babassu is considered the largest native oil resource worldwide and occurs naturally in Brazil and Colombia (Empresa Brasileira de Pesquisa Agropecuária 1984) and refers to three distinct genera of the family Arecaceae: *Scheelea*, *Attalea* and *Orbignya*, and the species *Orbignya phalerata* Mart. is the most common and widespread (Teixeira 2008). The area of occurrence of babassu is a transition zone between the rainforests of the Amazon Basin (northern region) and semi-arid lands of Northeast Brazil.

Babassu palm trees can reach 20 m in height, with the production of four bunches of fruits (drupes) per palm per season, and each cluster can provide 15–25 fruits (Teixeira 2008; Lorenzi 2010). Babassu palm trees start the production cycle between seven and ten years and end at 35, with a productivity of 2.2 to 15.6 tons of fruit per ha/year (Nogueira and Lora 2003).

Concerning the current availability of babassu nut residues in Brazil, Dias et al. (2012) estimated, based on a kernel production of 106,055 tons (Instituto Brasileiro de Geografia e Estatística – IBGE 2010), a total of 1,409,016 tons. On the other hand, (Teixeira 2008) estimated a Brazilian potential of 6.8 million tons of fruits/year; Maranhão is the state with the highest potential (92%), since improvements on the process of silvicultural operation are made.

The babassu nut residue (or shell) consists of all three constituent layers of the fruit (epicarp, mesocarp and endocarp). These layers correspond to approximately 93% of the total fruit (Dias et al. 2012; Empresa Brasileira de Pesquisa Agropecuária 1984; Emmerich and Luengo 1996). Therefore, for each ton of babassu nut, there are 930 kg of residues. However, despite this large supply of lignocellulosic residue, most of this biomass is inappropriately discarded (Dias et al. 2012), which may bring negative impacts to the environment.

Given the considerable supply of babassu nut residues in the Brazilian territory and their social importance to extractive communities (Dias et al. 2012; Teixeira 2008; Porro et al. 2011) research related to their proper use, where the analysis of the energy potential of this biomass is one of the feasible options, becomes fundamental. This analysis aims to contribute to their proper and efficient use, either in the production of heat or steam boilers, or in charcoal production for the steel industry, bio-oil, gas fuels, second-generation ethanol and cooking food.

Given the above, the objective of this study was to evaluate the potential for bioenergy use of babassu nut residues (epicarp, mesocarp and endocarp), especially considering direct combustion and charcoal production, through the analysis of their chemical, physical, energy and thermal characteristics.

## Methods

### Collection site and sampling of babassu nut biomass

**Figure 1:**
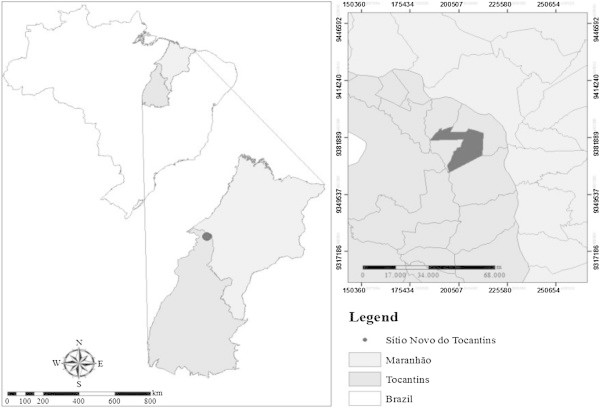
**Collection site of babassu nut residues.**

The three layers constituting the babassu nut were used together, i.e. epicarp, mesocarp and endocarp. The material was collected in the rural area of the municipality of Sítio Novo do Tocantins, in the state of Tocantins, Brazil (Figure 1) and is obtained from the extractive exploitation by local communities. Babassu nut shell comes from manual breaking. The biomass had about 10% moisture on a dry basis.

The collection site has a population of 9,148 inhabitants, an area of 324 km^2^, is located in the far north of the state, in the Tocantins River valley, in the region known as “bico do papagaio” (Figure 1) and is characterized by having dense babassu trees (Instituto Brasileiro de Geografia e Estatística – IBGE 2013).

### Morphological characterization of babassu nut fragments

A LEO EVO 40 XVP Zeiss scanning electron microscope was used, and images were obtained through secondary electrons, with magnifications of 37, 40 and 100x. The working distance (WD) considered was 9, spot size of 720 or 5.5 Kcps.

A representative fragment of the analyzed biomass, from which a 1.5 cm × 1.5 cm × 1.0 cm (2.25 cm^3^ volume) sample was removed, was used to observe the various layers of babassu nut (epicarp, mesocarp and endocarp). In order to avoid charging effects in the microscope chamber, the sample was subjected to metallization by sputtering with the deposition of a gold film on its surface.

Additionally, images of babassu nut fragments were obtained in natural scale, through a digital camera in order to demonstrate the typical morphological characteristics of the residual biomass analyzed.

### Chemical characterizations: molecular and elemental

For the chemical, energy and thermal characterization of the analyzed biomass, a 1 kg representative sample of the collected batch was removed. The material was processed in a hammer mil, homogenized, and was subsequently classified in 40, 60 and 200 mesh sieves. Energy and chemical tests were performed in quadruplicate.

The quantification of the amount of extractives was performed using the biomass fraction retained between the 40 and 60 mesh sieves, according to the standard NBR 14853 (Associação Brasileira de Normas Técnicas – ABNT 2010a). A Soxhlet extractor was used and the samples were subjected to a sequence of toluene-ethanol (2:1, 5 hours) ethanol (4 hours) and warm water (2 hours).

The determination of the insoluble lignin content was performed using the procedure described in the standard NBR 7989 (Associação Brasileira de Normas Técnicas – ABNT 2010b). The samples used were about 1 g, free of extractives, and the solvent used was 72% sulfuric acid, kept cold. The content of soluble lignin in sulfuric acid was determined by spectrophotometry and the equation described by Goldschimid was used (Goldschimid 1971). The total lignin content was considered as the sum of soluble and insoluble lignin.

The holocellulose content was obtained by difference in relation to other chemical and mineral constituents of biomass.

The elemental analysis was performed on an Elementar Vario Micro Cube universal Analyzer for the quantification of carbon, hydrogen, nitrogen and sulfur contents in relation to the dry mass of babassu nut residues. The samples retained between the 60 and 200 mesh sieves were used, in the same manner as used by (Protásio et al. 2013a). The oxygen content was determined by difference (Equation 1) (Bech et al. 2009; Protásio et al. 2013a). Based on the contents of the elemental constituents, the ratios N/C, H/C and O/C were obtained, as well as the molar ratios and the empirical formula of biomass.1

Where *O* is the oxygen content (%); *C* is the carbon content (%); *H* is the hydrogen content (%); *N* is the nitrogen content (%); *S* is the sulfur content (%) and *A* is the ash content (%).

### Immediate chemical composition and ash characterization

The immediate chemical analysis of biomass was performed to quantify the levels of volatiles and ash and, by difference, fixed carbon, according to the guidelines of ASTM D 1762–84 (American Society for Testing Materials – ASTM 2007).

Aiming for the quantification/qualification of the chemical elements present in ash, the energy dispersive X-ray analysis was performed in a Quantax X Flash 5010 Bruker machine, coupled to a LEO EVO 40 XVP Zeiss scanning electron microscope. The sample fractions retained on the 60 mesh sieve were mounted on two metallic stubs in a Union CED 020 carbon evaporator.

In the images obtained for each stub eleven random points were analyzed, and the arithmetic mean of the values found was subsequently characterized. In order to characterize ash, the chemicals were normalized to 100% and the molecular weight of the oxides (NiO, P_2_O_5_, F_2_O_3_, Cr_2_O_3_, Al_2_O_3_, MgO, CaO, K_2_O, and SiO_2_) was used to estimate the percentage of oxides in relation to total ash.

### Physical and energy characterizations

For the determination of the basic density of babassu nut residues, 31 fragments were randomly taken from the batch collected. The method of immersion in water was used, according to the guidelines of NBR 11941 (Associação Brasileira de Normas Técnicas - ABNT 2003). The dry mass of each fragment ranged from 10 to 40 grams, and the average was 24 grams.

For the obtention of the higher heating value (HHV), a digital C-200 IKA calorimeter was used, according to the procedures described in ASTM E711-87 (American Society for Testing Materials – ASTM 2004). The samples for the determination of HHV were classified in 40/60 mesh sieves, and the fractions of samples retained on the 60 mesh sieve, which were oven dried at 103 ± 2°Cuntil constant weight, were used in the test. The lower heating value (LHV), on a dry basis, was estimated using Equation 2.2

Where *LHV* is the lower heating value (kcal kg^−1^); HHV is the higher heating value (kcal kg^−1^) and *H* is the hydrogen content (%).

The energy densities were obtained by multiplying the HHV and LHV by the average basic density of babassu nut shell, as performed by (Protásio et al. 2013a) for other lignocellulosic materials.

### Thermal characterization: thermogravimetric analysis and differential thermal analysis (DTA)

For the thermogravimetric analysis and differential thermal analysis, the granulometric fraction that passed through the 200 mesh sieve was used. For this analysis, a SHIMADZU DTG-60H thermal analyzer was used.

The sample of about 4 mg was subjected to a temperature gradient ranging from room temperature to 1000°C with a heating rate of 10°C min^−1^, using a 50 mL min^−1^ nitrogen flow. Using the first derivative of the TG curve (DTG), which determines the mass loss *versus* temperature, it was possible to identify the rate of mass loss per second and the distinct pyrolysis stages.

## Results and discussion

### Morphological characterization of babassu nut fragments

**Figure 2:**
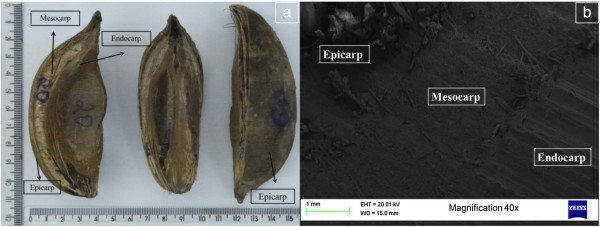
**Babassu nut fragments (a) and image obtained by scanning electron microscopy (SEM) (b).**

**Figure 3:**
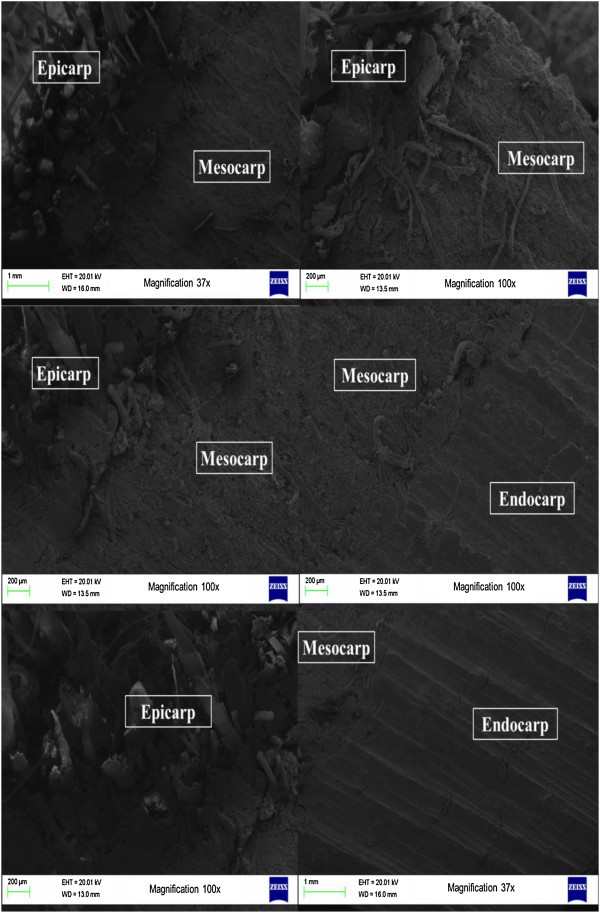
**Images obtained by scanning electron microscopy (SEM).**

The morphological aspects of the analyzed babassu nut fragments can be seen in Figures 2 and 3. The babassu fruits showed a differentiated morphology, which was approximately 9 cm long and a coefficient of variation of 8.95%.

Babassu nut fragments are formed by three distinct layers: a) the outer one, which is fibrous and thin (epicarp); b) the intermediate one, which is fibrous, with a high starch concentration (mesocarp) and c) the internal one, which is woody and very resistant (endocarp), in which kernels are inserted (Dias et al. 2012; Empresa Brasileira de Pesquisa Agropecuária 1984; Teixeira 2008; Nogueira and Lora 2003; Emmerich and Luengo 1996; Teixeira and Carvalho 2007; Teixeira 2005).

Generally, 12% of the fruit correspond to the epicarp, 23% to the mesocarp, 58% to the endocarp and 7% to kernels (Emmerich and Luengo 1996). Due to the chemical aspects, such as lignin and carbon contents, as well as to the physical aspects, such as density, the endocarp is the most important fruit component in charcoal production (Teixeira 2008).

The mesocarp, for being basically comprised of starch, has a high content of volatiles and low contents of fixed and elemental carbon (Teixeira 2008), which implies in a material with a low thermal stability that can considerably reduce charcoal and fixed carbon yield.

By analyzing the images obtained (Figure 3), it is possible to observe that the babassu endocarp presents a less porous, more lignified and dense aspect, at the expense of the mesocarp, which has a more porous structure. Thus, it is possible to infer that the density of the babassu nut is predominantly due to the presence of the endocarp and the carbonization yield will be higher, the smaller the amount of mesocarp and the greater the amount of endocarp in the fruit shell. Furthermore, it is expected that the fixed carbon yield of the endocarp is higher than the other constituents of the fruit, precisely due to its higher content of elemental carbon (Teixeira 2008).

### Chemical characterizations: molecular and elemental

**Figure 4:**
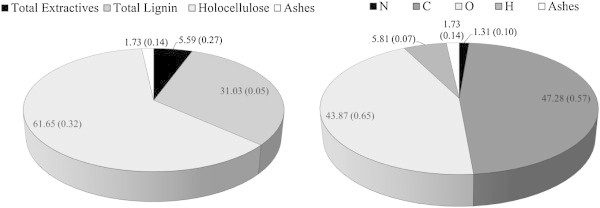
**Molecular and elemental chemical composition and ash content (% dry basis) of babassu nut residues (figures in brackets refer to the standard deviation).**

The knowledge of the chemical and molecular composition (Figure 4) is essential in the evaluation of the energy potential of a fuel. Through the contents of the elemental chemicals energy conversion processes, such as calculations related to the volume of air required for combustion and the amount of generated gases, can be analyzed, as well as enthalpy, exergy and heating value of the fuel (Nogueira and Lora 2003; Bilgen and Kaygusuz 2008).

Knowing the levels of nitrogen and sulfur, it is possible to estimate the pollution potential and the environmental impact related to the energy use of biomass. It is known that high contents of N and S are undesirable, because they contribute little in the heating value of plant biomass and, during the complete combustion of the material, these elements are almost totally converted into toxic oxides (NO_x_ and SO_x_) and can promote the formation of acid rain and soil acidification, as well as corrosion of equipment for energy conversion (Demirbas 2004a; Obernberger et al. 2006; Bilgen and Kaygusuz 2008; Huang et al. 2009; Kumar et al. 2010).

García et al. 2012 reported that SO_2_ emissions are negligible in biomass fuels. In the analyzed residues, only traces of this element were detected, which is a great advantage for the energy use of babassu nut residues, especially for charcoal production, since sulfur is a contaminant of pig iron and affects its mechanical properties.

The nitrogen content of babassu nut biomass was higher than that reported in the literature for sugar cane bagasse (N: 0.50%) (Paula et al. 2011; Protásio et al. 2013a) and wood of *Eucalyptus* clones (N: 0.07 to 0.25%) (Protásio et al. 2013a,[b]; Neves et al. 2011, 2013; Reis et al. 2012; Santana et al. 2012).

However, other lignocellulosic residues used for energy generation in Brazil, such as the residues from the processing of coffee beans and corn harvest present contents of N + S equal to 2.3% (Protásio et al. 2012a,[b]; Protásio et al. 2013a) and 2.2% (Protásio et al. 2013a), respectively, which are higher than those found for babassu nut biomass in approximately 72%.

Differences among the percentages of nitrogen reported in the literature and babassu nut residues may be explained by the different soil conditions, water stress, metabolism and physiology of each species. The N uptake in the soil is predominantly performed in the form of nitrate, so the local edaphoclimatic conditions are prevalent in the percentage of this element in plant biomass.

It is worth noting that coal, widely used in thermal power plants throughout the world, presents nitrogen and sulfur contents of up to 2.12% and 6.29%, respectively (Ward et al. 2008), which are much higher than the ones presented by babassu nut residues, and this fact reinforces the advantage of use of this biomass as an energy source. Demirbas 2001a reported that biomass combustion produces 90% less sulfur than coal.

As for the remaining elemental constituents, the high proportion of O, compared to C and H, typically reduces the heating value of the fuel, due to the low exergy contained in carbon-oxygen bonds, when compared to the energy in carbon-carbon or carbon-hydrogen bonds (Sheng and Azevedo 2005; Bilgen and Kaygusuz 2008; Huang et al. 2009; Demirbas 2004b; Kumar et al. 2010; Protásio et al. 2011). The results are in agreement with those obtained by Protásio et al. 2013a for sugar cane bagasse (C: 46.8%, H: 6.3% and O: 45.3%) and *Eucalyptus* wood waste (C: 48.2%, H: 6.4% and O: 45.0%) and show that the residual biomass of babassu nut presents a significant potential for bioenergy use, especially for direct combustion, aiming for the generation of heat and electricity.

For the charcoal production from babassu nut residues to meet the specifications of steel plants, the contents of biomolecules constituents of biomass must be considered. The lignin macromolecule has a predominantly aromatic and three-dimensional matrix, consisting of phenylpropane units, and therefore has a higher thermal stability than the carbohydrates in plant biomass, i.e. has less mass loss during pyrolysis (Sharma et al. 2004; Yang et al. 2007; Gani and Naruse 2007; John and Thomas 2008; Nakamura et al. 2008; Burhenne et al. 2013; Nowakowski et al. 2010). Yang et al. 2007 reported that the solid residue from lignin pyrolysis was high (~46 wt%) to the final temperature of 900°C.

Furthermore, lignin correlates with the heating value (Demirbas 2001b; Protásio et al. 2012b) and with the fixed carbon content (Demirbas 2003), due to its higher carbon content and lower oxygen content (Nowakowski et al. 2010), as well as due to the C = C bonds with a higher binding energy (Atkins and Jones 2006).

In the literature, it is possible to find average lignin contents for wood of *Eucalyptus* clones at ages 34, 42, 68 and 90 months: 29.6%, 31.4%, 29.8% and 30.0% and; holocellulose contents of: 66.7%, 64.2%, 66.7% and 65.47%, respectively (Neves et al. 2011; Santana et al. 2012; Protásio et al. 2013b; Pereira et al. 2012).

Despite the similarity of the total lignin content in *Eucalyptus* wood reported in the literature with the analyzed babassu biomass, the quality of lignin differs considerably between eudicotyledonous and monocotyledonous angiosperms, and babassu is classified in the second group. The lignin of leafy vegetables presents higher amounts of the precursor unit of syringyl (*trans*- sinapyl alcohol) than guaiacyl (*trans*-coniferyl alcohol) in variable proportions (Del Río et al. 2005; Nunes et al. 2010). The lignin of monocotyledonous angiosperms is composed of syringyl, guaiacyl and coumaryl (*trans*-p-coumaric alcohol) units, and the syringyl unit is present in smaller amounts (Nowakowski et al. 2010).

The basic difference between the types of lignin is the amount of methoxyl groups and the amount of C-C bonds on the aromatic ring. The absence of methoxyl groups in the structure of coumaryl lignin (formed by the *trans*-p-coumaric alcohol) enables a higher lignin condensation, due to an increase in C-C bonds with another coumaryl unit. Thus, it is expected that babassu nut residues enable a higher yield and quality of charcoal, when compared to *Eucalyptus* wood, which is widely cultivated in Brazil for this purpose.

Based on the elemental chemical composition of the analyzed biomass (Figure 4), the ratios N/C (0.03), H/C (0.12) and O/C (0.93) were determined, as well as the empirical formula of the analyzed babassu nut residues: CH_1.48_O_0.70_ N_0.02_. The smaller the ratios O/C and N/C, the better the thermal properties of the fuels.

The highest H/C ratio may correspond to the presence of a greater amount of aliphatic compounds (Cao et al. 2011), rather than aromatic compounds (such as extractives and lignin). This can promote a decrease in the heating value of the biomass fuel, as well as a decrease in the carbonization yield. Protásio et al. 2013b found a H/C ratio of 0.13 to *Eucalyptus* wood clones at 42 months old, that is, greater than that found for the babassu nut biomass (0.12), corresponding to a difference of 8.3%. This result reinforces the earlier discussion on the qualitative differences of lignin of babassu residues.

Extractives are a group of heterogeneous substances (Telmo and Lousada 2011) and the content in biomass is an important aspect in bioenergy production (Vargas-Moreno et al. 2012) since they are highly flammable compounds (Poletto et al. 2012), with low molecular weight, low activation energy (Guo et al. 2010) and are related to plant defense mechanisms.

Guo et al. 2010 stated that extractives decompose at low temperatures (150-600°C) and decrease the activation energy of combustion or pyrolysis. Thus, it can be assumed that the presence of extractives in biomass can be critical in the initial reactions of combustion and pyrolysis. Given the above, it is expected that the extractives may promote an increase in the heating value of biomass (Telmo and Lousada 2011).

For the residues from the processing of coffee beans, Protásio et al. 2013a reported an average total extractives content of 8.6% and Protásio et al. 2013c, working with the pyrolysis of a similar material, attributed the high content of extractives to the higher material degradation at lower temperatures, due to their higher volatility and flammability.

For the wood of *Eucalyptus* clones, at ages 34, 42, 68 and 90 months, some authors reported the following average levels of total extractives: 3.10%, 4.16%, 3.28%, 4.33% , respectively (Neves et al. 2011; Santana et al. 2012; Protásio et al. 2013b; Pereira et al. 2012), which were lower than that observed for the babassu nut biomass. Therefore, in order to burn this residue in boilers, gasifiers or other energy conversion mechanisms, the presence of extractives in babassu nut can facilitate the ignition of biomass, due to the decrease in its activation energy.

### Immediate chemical composition

**Figure 5:**
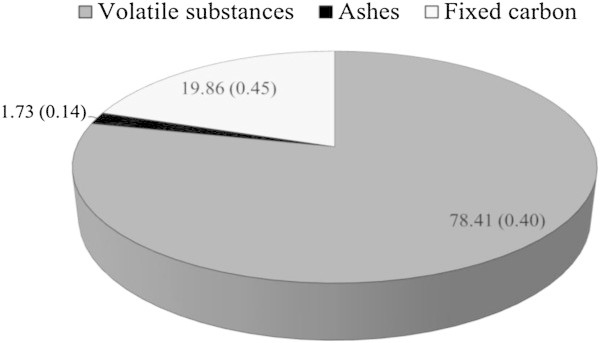
**Immediate chemical composition (% dry basis) of babassu nut residues (figures in brackets refer to the standard deviation).**

The knowledge of the immediate chemical composition (fixed carbon, volatiles and ash) (Figure 5) is essential to estimate the degree of biomass combustion, especially if the fuel is used to generate heat, steam or electricity, as well as for cooking food.

The volatile materials from biomass fuels are a complex mixture of gases and liquids derived from the thermal decomposition of molecular chemicals and usually consist of H_2_0, H_2_, CO, CO_2_, CH_4_ and tar, which is a complex mixture of condensable hydrocarbons (Yang et al. 2007; Amutio et al. 2012) and promotes an easy and rapid combustion of biomass (Werther et al. 2000).

When biomass is subjected to high temperatures, the volatilization of these constituents, which are mixed with the oxygen in the air, occurs and promotes homogeneous combustion reactions (Nogueira and Lora 2003). These reactions, especially ignition, are important in the early stages of pyrolysis and combustion. Knowledge of the content of volatile materials is essential for the planning of furnaces and amounts of air required for the smooth flow of gases and proper combustion of biomass in energy conversion systems (García et al. 2013).

Protásio et al. 2013a found contents of volatiles ranging from 68.3% for rice husk to 86.7% for *Pinus* shavings. The authors attributed these differences to the molecular chemical composition of biomass and found that these fuels showed the lowest (16.18 MJ kg^−1^) and the highest (20.37 MJ kg^−1^) heating values. Thus, the positive influence of volatiles on the heating value and on biomass reactivity has been demonstrated in the literature (Sheng and Azevedo 2005; Akkaya 2009; García et al. 2013). Generally, the contents of volatiles in biomass fuels range from approximately 70% to 87% (Protásio et al. 2012a; Protásio et al. 2013a; Protásio et al. 2013c; García et al. 2013), which is in agreement with the results found in this study.

Fixed carbon indicates the fraction of non-volatile organic matter, but may contain oxygen and hydrogen (Parikh et al. 2007). Thus, the higher the fixed carbon content, the slower the biomass combustion within the apparatus for energy conversion, such as stoves or boilers, and more thermally resistant will be the biomass (Protásio et al. 2013c). Protásio et al. 2013a found fixed carbon contents of 14%, 13% and 13.1% in Brazilian lignocellulosic residues from *Eucalyptus* wood, pine wood and sugar cane bagasse, respectively, which are lower than the one observed for babassu nut biomass. This result can be attributed to the quality and quantity of lignin in babassu residues, since this macromolecule is correlated with the fixed carbon content of biomass (Demirbas 2003).

Thus, the slower burning of babassu nut residues can be advantageous for cooking food, since burning appliances (stoves) widely used in the North and Northeast of Brazil by rural communities have a low efficiency in the use of the heat produced by the oxidation of biomass, due to the lack of technology for refining and improving the performance of the stoves. It is known that about 11.0% of the biomass produced in Brazil is intended for residential consumption (Empresa de Pesquisa Energética – EPE 2012).

As for the ash content in fuels, high levels are undesirable for the direct use of biomass in power generation, as well as for charcoal production, because minerals do not participate in the oxidation of fuel. Ash reduces both heating value and exergy, decreases fuel flammability and heat transfer, in addition to increasing the corrosion of equipment and causing power losses by the heating of mineral oxides (Bilgen and Kaygusuz 2008; Akkaya 2009; Tan and Lagerkvist 2011; Bustamante-García et al. 2013).

Some Brazilian lignocellulosic residues, which may be useful for bioenergy generation, present a higher ash content than that found for the residual biomass of babassu nut, such as residues from the processing of coffee beans (4.9%), maize harvest (6.8%), rice husk (16.8%) (Protásio et al. 2013a) and sugar cane bagasse (11.3%) (Bragato et al. 2012). Coal, seen as an essential fuel in the energy matrix of many countries, has a high ash content, ranging from 8.1% to 21.4% (Ward et al. 2008; Bragato et al. 2012).

Based on these results, the potential of the bioenergetic use of babassu nut becomes evident; in addition, based on the characterization of mineral oxides present in ash (Table 1) new products can be obtained or the degree of fouling in the equipment can be estimated.

**Table 1:** **Estimates of the oxides present in the ash of babassu nut residues (% dry mass)**

Babassu nut residues	K_2_O	SiO_2_	Al_2_O_3_	Cr_2_O_3_	CaO	P_2_O_5_	Fe_2_O_3_	MgO	NiO	Total
	0.36	0.33	0.27	0.18	0.17	0.15	0.14	0.12	0.01	1.73

Werther et al. 2000 reported that the biggest problem related to the burning of agricultural residues is the low melting temperature of ash, especially by the presence of K_2_O. According to Stern and Gerber (2004), potassium and calcium define the melting temperature of ash and, the smaller the K_2_O/CaO ratio, the higher the melting temperature of ash. Generally, the K_2_O/CaO ratio ranges from 0.2 to 0.8 for wood ash (Stern and Gerber 2004), which is higher than that found for babassu nut residues. However, it should be noted that the analyzed babassu biomass showed a low ash content compared to various agricultural residues reported in the literature and to coal, as discussed earlier.

The high content of K_2_O found for babassu nut shell, compared to other mineral oxides, may be due to the importance of potassium for biomass production and maintenance of osmotic balance in plants. This element also provides the plant with resistance to adverse conditions, such as low water availability and high temperatures.

### Physical and energy characterizations

The higher and lower heating values, the energy densities and the basic density of babassu nut residues are in Table 2, as well as some results of the main plant biomasses used for energy purposes in Brazil.

**Table 2:** **Higher heating value (HHV), lower heating value (LHV), energy densities base on HHV (ED**_**HHV**_ **) and LHV (ED**_**LHV**_ **) and basic density (BD)**

	Babassu nut residues^a^	Sugar cane bagasse^b^	Residues from the processing of coffee beans^b^	Wood of ***Eucalyptus*** sp. clones (42 months)^c^
BD (kg m^−3^)	1,273^(81)*^	104	249	521
HHV (MJ kg^−1^)	18.47^(0.10)^	18.89	19.29	19.16
ED_HHV_(GJ m^−3^)	23.51^(0.13)^	1.96	4.80	9.99
LHV (MJ kg^−1^)	17.16^(0.09)^	17.32	17.71	17.74^**^
ED_LHV_(GJ m^−3^)	21.84^(0.12)^	1.80	4.41	9.25^**^

^a^:observed in this study; ^b^:values obtained by (Protásio et al. 2013a); ^c^:average values obtained by Protásio et al. 2013b. *Figures in brackets refer to the standard deviation; **: average values calculated based on the information by Protásio et al. 2013b and the same methodology used in this study.

The use of the lower heating value in these calculations is important because it does not include the latent heat of water condensation present in combustion products, that is, it is the actual amount of power produced by the complete combustion of the material. The higher calorific value is important to make comparisons between different values of biomass (Protásio et al. 2013a) and represents the maximum amount of energy that can be released by the fuel (Nogueira and Lora 2003; Friedl et al. 2005).

It is possible to observe the similarity of the higher heating value (HHV) and the lower heating value (LHV) in the babassu nut shell with other biomasses used in Brazil as bioenergy sources. It is possibly due to similarities in the chemical composition, especially in the contents of carbon, hydrogen and oxygen of these fuels, as discussed earlier.

However, the superiority of the basic density in babassu nut shell is a great advantage, because it maximizes energy densities (HHV or LHV basis), i.e. the analyzed biomass has more energy stored per unit volume compared to the sugar cane bagasse, to the residues from the processing of coffee beans and to the wood of *Eucalyptus* spp. clones (Protásio et al. 2013a; Protásio et al. 2012a). Therefore, a greater efficiency and economic viability in the transport of babassu nut are expected, if that biomass needs to be used outside the producing regions to generate heat or electricity, as well as for charcoal production.

In this context, analyzing the information from the literature regarding *Eucalyptus* wood (Protásio et al. 2013b), which is widely used in Brazil for charcoal production of steel use, it is possible to observe that the babassu nut shell will certainly provide charcoal with high apparent density and mechanical resistance, which can be used in steelmaking as a direct substitute for metallurgical coke, because it solves two constraining factors for the use of wood charcoal: low density and low compressive strength, as observed by Emmerich and Luengo 1996. It is known that the higher the density of plant biomass, the greater the density and strength of charcoal in blast furnaces.

### Thermogravimetric analysis and differential thermal analysis

**Figure 6:**
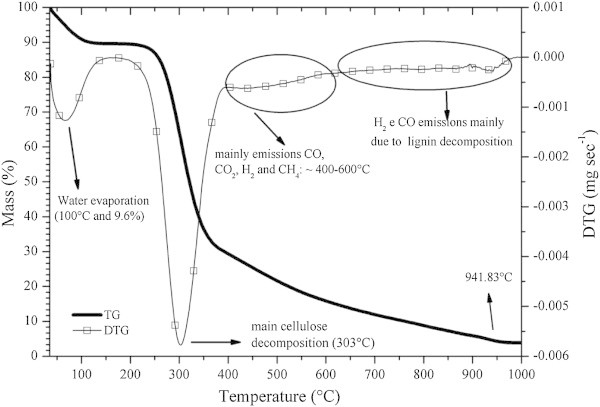
**Mass loss** ***versus*** **temperature (TG curve) of babassu nut residues under continuous nitrogen flow.**

Plant biomass reacts in three distinct stages during pyrolysis, as can be seen in Figure 6. Some endothermic and exothermic reactions occur with the release of CO, CO_2_, CH_4_, H_2_, and certain organic compounds of low molecular weight (C_n_H_m_) from the decomposition of the main constituents of plant biomass: cellulose, hemicellulose and lignin (Yang et al. 2007; Cheung et al. 2011; Amutio et al. 2012; Abnisa et al. 2013); pyrolysis is a predominantly endothermic process (Cheung et al. 2011).

**Figure 7:**
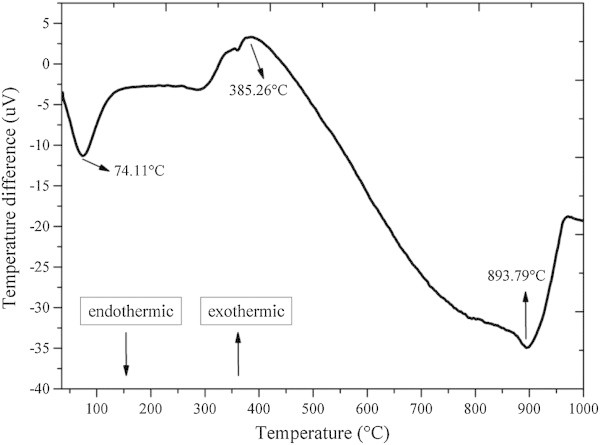
**Differential thermal analysis (DTA) of of babassu nut residues under continuous nitrogen flow.**

Exothermic reactions involve biomass cracking in small fractions during the initial stage of pyrolysis at low temperatures. As the temperature increases, some primary products are vaporized and produce secondary products, characterizing endothermic reactions (Cheung et al. 2011; Abnisa et al. 2013), as can be seen in Figure 7.

In stage I, the evaporation of water of the biomass (drying) occurs, and in the second stage, the mass quickly decreases due to the volatilization of cellulose and hemicellulose (holocellulose) and then, during the third stage, the mass decreases less intensely mainly due to the thermal decomposition of lignin and its products. This is because hemicelluloses are degraded between 220°C and 315°C, cellulose between 275°C and 350°C, lignin between 150°C and 900°C and extractives between 150°C and 600°C (Kim et al. 2006; Yang et al. 2007; Gani and Naruse 2007; Guo et al. 2010; Poletto et al. 2012).

Between 200-400°C, there is the formation of organic hydrocarbons of low molecular weight (C_2_H_6_ and C_2_H_4_), and a mixture of acids, aldehydes (C = O), alkanes (C-C) and ethers (C-O-C) (Yang et al. 2007; Amutio et al. 2012) resulting mainly from the decomposition of holocellulose.

Although lignin loses mass at lower temperatures, its loss rate of is much lower than the other chemical components of plant biomass (Burhenne et al. 2013). Furthermore, the decomposition of the chemical constituents of biomass does not occur separately, but some compounds are produced mainly by the breaking of a certain molecule of biomass (Yang et al. 2007).

It is possible to observe an initial mass loss (stage I) for the pyrolysis of babassu nut, corresponding to an evaporation of water of 9.6%, followed by an intense mass loss (60.2%) mainly attributed to holocellulose (stage II). The onset and endset temperatures of this stage were 280°C and 342°C, respectively. The peak of maximum mass loss was found at 303°C and it is lower than those reported by Poletto et al. 2012 and Protásio et al. 2013c for *Eucalyptus* wood, at 364°C and 354°C, respectively.

The presence of extractives (components of low molecular weight) in babassu nut residues (Figure 4) in higher amounts than in *Eucalyptus* wood can promote biomass flammability at lower temperatures, due to their higher volatility and, then, speed up the thermal degradation process, as well as the presence of a lower crystalline cellulose content (Grønli et al. 2002; Guo et al. 2010; Poletto et al. 2012; Protásio et al. 2013c; Shebani et al. 2008).

In addition, the starch present in the mesocarp, approximately 70% (Nogueira and Lora 2003), may also have contributed to the degradation of babassu nut biomass at lower temperatures. Teixeira 2008 found a high content of volatile material in the mesocarp of babassu nut (~ 95%) and stated that this component of the fruit provides a very quick burning with a low carbonization yield, at the expense of the endocarp, which presented lower emissions of volatile materials (83.40%) and a higher fixed carbon content (15.16%), being more suitable for burning and carbonization, in relation to the other constituents of the fruit.

This explains the lower value found for the temperature of maximum mass loss for babassu nut biomass, compared to the *Eucalyptus* wood found in the literature (Poletto et al. 2012; Protásio et al. 2013c), since the mesocarp presents a relevant participation in babassu nut (Figures 2 and 3).The third stage of thermal degradation, mainly due to the decomposition of lignin and of the gases formed during pyrolysis, showed a mass loss of 25.3%, resulting thus in a total mass loss of 95.1% at 1000°C. The onset and endset temperatures in this stage were 417°C and 703°C, respectively, and show the high thermal stability of the lignin present mainly in the endocarp, because the mass loss in stage III was lower in approximately 138% compared to stage II. In Figure 6, a small peak of mass loss at 941.83°C, characterized by an endothermic reaction (Figure 7), is also observed. This result can be attributed to the degradation of lignin and corroborates the earlier discussion on the differentiated quality of lignin in the babassu nut shell.

Considering the most used range of wood carbonization in Brazil (400-500°C) the total cumulative mass loss observed in the thermogravimetric essay for babassu nut shell was approximately 77%. As for *Eucalyptus* clones, Santos et al. (2012) observed a total mass loss of 85% at this temperature range, that is, 10.4% higher than the result of this study. Similarly, Protásio et al. 2013c observed a total mass loss of 82% up to 500°C for *Eucalyptus* sawdust. This highlights the carbonization potential of babassu nut shell, considering the quality and yield of charcoal which, combined with the high density of this biomass, will surely provide a charcoal that meets the specifications of blast furnaces in steel plants.

As for the gases produced during pyrolysis, CO_2_ presents a maximum release peak between 450°C– 500°C and decreases significantly with the increase in the pyrolysis temperature, while the concentration of CO increases (Amutio et al. 2012). This occurs because CO_2_ is produced mainly by the release of carboxyl groups (R–COOH) present in hemicelluloses which, in turn, show thermal decomposition at lower temperatures (Kim et al. 2006; Yang et al. 2007; Amutio et al. 2012).

The cellulose molecule is the main responsible for the formation of CO during pyrolysis, since it presents a greater amount of carbonyl groups. The maximum formation peak of carbon monoxide is around 450°C (Yang et al. 2007).

The release of H_2_ begins at temperatures higher than 400°C with a more intense volatilization from 600°C (Yang et al. 2007; Amutio et al. 2012) on, and lignin is the main chemical compound responsible for the formation of gas fuel during pyrolysis (Yang et al. 2007). This would explain the presence of mass loss in the range between 864 and 1000°C. The production of CH_4_ occurs significantly at temperatures from 500 to 600°C and, as well as the production of H_2_, can be associated to the lignin aromatic rings and to the O-CH_3_ functional groups (Yang et al. 2007).

Thus, the mass loss (~14%) lying in the range from 400°C to 600°C may be related to the volatilization of CO, CO_2_, CH_4_ and H_2_ from pyrolysis, as well as other hydrocarbons of lower molecular weight (C_2_H_6_ e C_2_H_4_).

## Conclusions

Babassu nut residues presented a significant energy potential mainly due to their high energy density, compared to various biomasses commonly used for power generation in Brazil.

The results found show that the use of babassu shell as a bioenergy source in the direct production of either heat or electricity, can be highly feasible, given its chemical and thermal characteristics, combined with a low ash content.

The babassu nut shell showed high basic density and suitable lignin content for the sustainable production of bioenergy and charcoal technically capable of replacing coke in Brazilian steel plants. This can contribute decisively in the economic development of extractive communities, who survive from the babassu nut collection, by marketing a product with higher added value.
